# Controlled Substance Waste: Concerns, Controversies, Solutions

**DOI:** 10.7759/cureus.22564

**Published:** 2022-02-24

**Authors:** Frank Breve, Jo Ann K LeQuang, Lisa Batastini

**Affiliations:** 1 Department of Pharmacy, Temple University, Philadelphia, USA; 2 Research, NEMA Research, Inc., Naples, USA; 3 Pharmaceutical Law and Drug Diversion, Mid Atlantic PharmaTech Consultants, LLC, Ventnor City, USA

**Keywords:** regulations, disposal of controlled substances, drug diversion, environmental protection, pharmacology

## Abstract

Hospitals, clinics, and organizations using controlled substances must have policies and procedures in place for disposing of these substances and to avoid potential drug diversion as well as environmental pollution. Challenging, particularly to hospitals, is the ability to dispose of the waste of any number of hundreds of products every day, some of which require specific handling and protocols for safety. Incineration might be appropriate but many hospitals and certainly smaller clinics lack the appropriate facilities. Clinics and facilities that use controlled substances must maintain adequate and detailed records, but individual healthcare systems impose their own specific requirements. Some, for example, require drug disposal to be witnessed. However, recordkeeping systems must be robust and frequently audited to prevent diversion. Most healthcare systems want to dispose of controlled substances in an environmentally responsible way but in addition to federal laws in the United States, most states have their own environmental agencies and may have local regulations. Navigating this system can be complex, and since all regulations are subject to change, it requires vigilance and expertise.

## Introduction and background

Despite the widespread prescribing of controlled substances in the United States (US), the nation lacks a uniform protocol for the handling and disposal of controlled substance waste [1]. Organizations that utilize controlled substances must have plans in place to effectively deal with such waste and its ultimate disposal, and in the lack of clear-cut guidance, it is important that these organizations develop sound, defensible, and robust programs on their own.

The network of official organizations that oversee the manufacture, regulation, distribution, and disposal of controlled substances is large and fragmented. Since the individual agencies do not always communicate effectively with each other, contradictory or impractical guidance is sometimes issued. A good example is that the US Food and Drug Administration (FDA) advises people to flush unused opioid analgesics down the toilet with its “flush list,” [2] while the US Environmental Protection Agency (EPA) advises against this practice because it can contaminate the water supply [3]. Manufacturers of many opioid analgesics, for example, advise end users to flush unused products [4,5], as they are more tightly regulated by the FDA than the EPA. For hospitals and other organizations, this may not be the case [6].

While pharmaceutical waste disposal at manufacturing plants typically involves a large quantity of one or a few products, most hospital pharmacies stock thousands of different pharmaceutical products, many used only in small quantities, and each of which has specific regulations governing its disposal. Nurses do not typically receive academic training in waste disposal. and experts in environmental concerns do not typically receive academic training in the various active agents in pharmaceutical products [7]. Of all of these substances, the proper wasting of controlled substances is likely the most important to the environment and public health and also one of the areas where there is considerable confusion. This is far from a matter that affects only hospitals as many organizations in the US buy, maintain, dispense, and must dispose of controlled substances (See Table 1).

**Table 1:** Organizations, entities, or areas that generate or handle controlled substance waste

Entity	Areas	Comments
Hospitals	Onsite pharmacy, operating rooms, recovery rooms, post-anesthesia care unit, intensive care units, nursing units	Includes academic teaching hospitals, general hospitals, community hospitals, specialty hospitals (cancer, rehabilitation, eye, psychiatric, etc.). Prescriptions written for end-user.
Acute care centers	Onsite pharmacy, clinic	Includes emergency rooms of hospitals. Prescriptions written for end-user.
Surgical centers	Operating rooms, recovery rooms, post-anesthesia care unit	Includes ambulatory care facilities. Prescription written for end-user
Long-term care facilities	Nursing units, medication room storage	Includes skilled nursing facilities, assisted-living facilities, elder or medical day care facilities.
Physician offices	Secured, locked cabinets	For on-site treatments. Prescription written for end-user.
Dental offices	Secured, locked cabinets	For on-site treatments. Prescription written for end-user.
Pharmacies	Controlled substance inventory stored in a safe, in secure locked cabinets, or interspersed among regular drug stock shelves	Stand-alone retail pharmacies, chains, supermarkets, retail outlets, as well as out-patient pharmacies working with a hospital or other clinical organization.
Veterinarians	Secured, locked cabinets	For on-site treatments. Dispensing for off-site use. Prescription written for end-user.
Medical researchers	Clinical trial investigators, Bench researchers	Controlled substance storage in narcotic safes or in secure, locked cabinets. For on-site research subjects.
Narcotic treatment programs	Inpatient treatment, outpatient treatment	Medication assisted treatment (methadone, buprenorphine, benzodiazepines).
Behavioral health facilities	Inpatient treatment, outpatient treatment	Controlled substances utilized for psychiatric and medical care of the patient.
Hospice	In-home or in-hospital units	Controlled substances used for comfort care, end of life care.
Law enforcement	Seizure of illicit drugs, public voluntary returns	Authorized collection receptacles. Collection for supervised destruction.
Government agencies	Veterans Administration, Department of Defense	Out-patient and in-patient pharmacies, controlled substance stock kept on nursing units. Prescriptions written for end-user.
Pharmaceutical supply chain	Manufacturers, Distributors, Sales Representatives	Warehouse storage and outsourcing. Transportation regulations which conform to chain of custody requirements. Samples for DEA-licensed practitioners
Reverse distributors	On-site removal and transfer for off-site destruction/incineration	May interface with transportation companies, transportation regulations
Waste management companies	On-site removal and transfer for off-site destruction/incineration	Licensed by DEA, subject to transportation regulations
Home patients	Considered end user, dispensed as per practitioner prescription	Home storage, (example: medicine cabinet)
Nurses at schools, universities, campus clinics	Medication cabinets	Controlled substances administered to children and students (example: Attention Deficit Hyperactivity Disorder)
Funeral homes and mortuaries	Funeral Director office, Preparation rooms	Controlled substances of deceased brought in by family members or identified in the embalming process (example: drug addicts)
Medical examiners	Autopsy rooms	Controlled substances identified on corpse during autopsy (example: drug addicts) or in personal belongings
Prisons and correctional facilities	On-site pharmacy, Dispensaries	Controlled substances used for inmate, inpatient treatments
Emergency medical services	Ambulances, Survival Flight	Controlled substances are supplied in kits for Emergency Medical Technician use
Marijuana dispensaries	On-site inventory, variety of marijuana products for sale	Classified as C-I controlled substances and technically illegal under Federal law
Academic institutions	Lab areas in Medical Schools, Pharmacy Schools, Dental Schools	Controlled substances are utilized for teaching exercises, experiments, and research activities
Municipal health clinics	On-site pharmacy	Controlled substances are somewhat utilized on-site for treatment. Prescriptions dispensed by on-site pharmacy
Urgent care centers	Some have on-site pharmacies	Limited prescribing on pain medication

## Review

Diversion concerns

While controlled substance diversion exists in the healthcare system, it has been challenging to objectively quantify [8,9]. This is exemplified by the fact that no one knows the true extent of drug diversion; estimates indicate that 10% or more of healthcare professionals have diverted controlled substances [10]. Thus, it has been difficult to issue definitive guidance in terms of how to prevent it [11]. For example, theft of controlled substances by healthcare personnel is likely a significant source of drug diversion, but the weak points in the healthcare system where such diversion could occur have not been clearly identified. In fact, hospital-based diversion may go unnoticed for long periods of time [12]. While there is a wide availability of practical recommendations to set up a system for minimizing the risk of diversion, there is a lack of practical recommendations relating to reducing the risk of diversion by people tasked with handling controlled substances as part of their job. It is unfortunate that many facilities fail to incorporate robust practices until they are cited for theft and diversion by regulatory authorities. Yet the consequences of such theft and diversion for hospitals and other healthcare systems can be enormous: financial penalties, reputation damage, liabilities associated with inadequate care, lack of employee productivity, regulatory penalties, and risks to both patients and healthcare professionals. Using a Failure Modes and Effects Analysis model, a study of four hospitals with 1242 inpatient beds mapped out a series of 10 major and 30 incremental steps in the custodial chain of controlled substances from ordering to confirmation of inventory to the return or disposal of unused controlled substances. This resulted in the identification of 24 points considered to be potential points of diversion, such as manual loading of requests, forgery of required signatures, failure to have controlled substance disposal witnessed, inventory inaccuracies, failure to reconcile reports with the offsite distributor, documentation of controlled substance disposal but without actual disposal, ordering more controlled substances than required, tampering with waste, and others [12]. These vulnerabilities may differ among healthcare systems, as individual hospitals may have specific protocols or processes that reduce risk at certain points. A survey of pharmacy directors at various US healthcare systems (n=140) reported on wide variation in practices among acute-care institutions for preventing diversion of controlled substances [13]. Thus, most hospitals and clinics are at risk for improper wasting of controlled substances leading to diversion but may not know it or be aware of what can be done to shore up their system.

Diversion may also occur in pharmacies, where billions of retail prescriptions are filled annually. Most prescriptions filled in the pharmacy are for outpatients and outpatient diversion occurs, but has not been thoroughly studied or quantified [11]. In fact, all of the entities and areas outlined in Table 1 can be subject to diversion activity at one time or another. One of the weakest links in the controlled substance chain of custody is the waste stream. It is relatively easy for a healthcare worker tasked with the disposal of a controlled substance to simply divert it for personal use and document that the drug had been wasted. For this reason, it is imperative that anti-diversion measures pay particular attention to how controlled substance waste is handled and disposed of. 

Regulatory concerns

The regulation of pharmaceutical waste is governed by overlapping regulations provided by municipal, state, tribal, and federal authorities. In some cases, states may impose more stringent regulations than federal regulations. Regulations vary among states (See Table 2).

**Table 2:** Federal, state, local, and tribal authorities may have specific rules regarding the wasting of controlled substances. This poses a challenge for the disposal of such substances as these authorities overlap somewhat, guidance is often revised and updated, and it can be difficult to adequately meet all relevant requirements. CWA: Clean Water Act; EPA: Environmental Protection Agency Adapted from:  Managing Pharmaceutical Waste: A 10-Step Blueprint for Healthcare Facilities In the United States [7]

Agency	Main Regulations and Overarching Goals	Specific Points of Interest
Environmental Protection Agency (EPA)	The Resource Conservation Recovery Act encourages disposal that has minimal environmental impact, promotes take-back events for ultimate users, and sets guidelines for healthcare facilities	Prohibits down-the-drain disposal
Department of Transportation	Requires appropriate containment and labeling of hazardous waste during transportation	Rules for appropriate labeling of waste products in transport
Drug Enforcement Administration (DEA)	Controlled Substances Act registers those permitted to handle controlled substance disposal	Controlled substances must be rendered non-retrievable, that is, their physical and/or chemical structure must be changed to the point that they are unusable
Occupational Safety and Health Administration	Controlling occupational exposure to hazardous drugs	Protects workers from dangerous contact with medical waste, including but not limited to controlled substances
State Environmental Protection Agencies	Environmental management on the state level	States may have different and more stringent requirements than the federal government
State Professional Licensing Boards	Oversight of professions that utilize controlled substances	Medicine, pharmacy, nursing, dentistry, veterinary, etc.
Local Publicly Owned Treatment Works	Operate under the CWA and EPA	Sets standard for pretreatment responsibilities and monitoring requirements
Food and Drug Administration (FDA)	Drug Chain Security Act	Traceability of controlled substances

At the federal level, the disposal of controlled substances is regulated by 21 Code of Federal Regulations (CFR) Part 1317, which states that they must be destroyed in such a way that they are non-retrievable and the method of destruction must be such that it prevents any diversion of the active-controlled substances for illicit purposes [14]. Incineration would meet this requirement, but many hospitals do not have suitable equipment for efficiently burning the drugs. Incineration may also pose certain environmental as well as occupational safety issues. Sewering is sometimes used, but it fails to meet the Drug Enforcement Administration (DEA) standard of rendering the drug non-retrievable, and it is discouraged by the EPA for environmental reasons [15]. The DEA stops short of recommending any specific destruction method.

The sewering of controlled substances is accepted by the FDA [16] and this is a common practice carried out at many facilities. In some cases, wastewater treatment plants must approve of such disposal, and such disposal of controlled substances through the sewage is prohibited outright in some areas [7]. In some instances, controlled substance waste can be transferred to an agency or organization that is authorized to destroy them. At least two witnesses must observe the transportation, transfer, and destruction of these controlled substances and sign off that they are rendered non-retrievable, if a third party is used. If on-site destruction of the controlled substances is done, the destruction must be witnessed by at least two employees who document that they personally witnessed the destruction and that the controlled substances were rendered non-retrievable [14]. However, there is no clear recommendation as to how the controlled substances are to be rendered non-retrievable.

Unused controlled substances in hospitals, clinics, or other settings are to be placed in a special waste receptacle that consists of an outer receptacle with a removable inner liner (much like a trash can with a plastic liner). While commercially available receptacles are available in the market, the DEA does not approve or endorse any specific products [17]. Furthermore, it should be noted that the cost and installation of these controlled substance collection receptacles may be expensive and the demands for continuous monitoring can also add to their expense [17]. The location of this controlled substance receptacle can likewise be problematic, in that it should be in a regularly monitored area, but should not be in a busy area where diversion could occur [14]. Continuous monitoring of the receptacle is advised but can be accomplished with closed-circuit video. In hospitals or other institutions with an onsite pharmacy, the controlled substance disposal receptacle need not be in the pharmacy and might be more conveniently placed at a nurses’ station or another area where such controlled substances are disposed.

Long-term care facilities may place a collection receptacle on-premises providing it is supplied by a retail pharmacy, hospital, or a clinic with an onsite pharmacy that has had its DEA registrations modified to become "collectors." Such entities are defined as registrants authorized to receive a controlled substance for the purpose of destruction [17]. Long-term care facilities are not authorized collectors, meaning they must rely on another organization to render their wasted controlled substances non-retrievable.

In some cases, unused controlled substances wind up the property of the ultimate user, such as a person who has leftover opioid analgesics at home following surgery. The DEA once ran local drug take-back programs in conjunction with community organizations or the police, but such programs are giving way to mail-back programs. Mail-back programs allow ultimate users of controlled substances to send back unused drugs in prepaid, pre-addressed packaging to an authorized collector, who then destroys the drug on receipt without inventorying the contents. This program may be vulnerable to diversion in that it relies on the ultimate user to return the drugs and the postal system to process, handle, and deliver them. Privacy regulations mean that it is prohibited for the authorized collector to ask for personal information from those participating in the program. If an authorized collector runs a mail-back program, the collector must have an onsite means of rendering the substances non-retrievable [17]. Ultimate users may not know about mail-back programs or may find them too much trouble. Many controlled substance manufacturers provide FDA guidance in their instructions for use [18,19], which recommends flushing the unused product despite the fact that the EPA does not support such disposal methods.

Recordkeeping concerns

Organizations, such as pharmacies, hospitals, clinics, and others, who handle and dispense controlled substances are required to maintain a system for keeping records about the use and destruction of these substances in order to document the controlled substance prescribed, the amount actually used, and the leftover amount destroyed. While specific steps are set forth for this kind of recordkeeping, there can be variations among organizations. Regulations require two witnesses of the disposal, although organizations may vary as to who these witnesses may be. In some cases, a pharmacist, nurse, or anesthesia care provider must serve as a witness and sign off that the wasted controlled substances have, in fact, been properly disposed of. Controlled substance waste is then transported for destruction or destroyed at the facility, with robust documentation to accompany these steps. Organizations must comply with regulations but may have some flexibility in setting up their recordkeeping protocols. As a result, some organizations develop fairly simple and straightforward rules for keeping their records.

While simplicity has its value, simple recordkeeping is easily circumvented; more complex recordkeeping systems might reduce diversion, but they are more difficult and time-consuming to implement, much less enforced [20]. The majority of hospitals, particularly the larger institutions, use decentralized automated dispensing machines (ADMs), which facilitate recordkeeping but are not entirely immune to diversion [21]. Thus, the recordkeeping process is vulnerable to diversion, particularly by those who can exploit the witnessing and sign-off requirements. There should be a quality assurance (QA) program in place that regularly assesses the wasting process to ensure that there are no deviations from policies and procedures, and that recordkeeping has not been compromised. 

Bedside wasting refers to the practice of disposing of unused controlled systems immediately. This is generally considered efficient and reduces the likelihood that controlled substances may be diverted, but bedside wasting can be burdensome in that it requires witnesses, documentation, and entry into recordkeeping systems. Such steps can interrupt the normal clinical workflow, particularly when the hospital or clinic is experiencing a surge of patients.

The amount of controlled substances that are bedside wasted may be substantial in some institutions. For example, if the pharmacy dispenses 5 mg midazolam for a patient but that patient uses only 3 mg, then 2 mg is wasted and 3 mg is counted as the net amount dispensed [22]. In a study of wasting of controlled substances, an average of 22.2% of drugs dispensed were wasted, with liquid formulations more likely to be wasted than oral products [22].

Environmental concerns

It is far from unusual for some healthcare organizations to dispose of unused controlled substances by flushing them down the drain and causing them to enter the municipal water supply. While concern about this practice has been raised with respect to numerous pharmaceutical products, even to the extent that specific protocols for the disposal of certain non-controlled drugs have been issued, controlled substances are often still sewered. Nurses and other hospital staff members tasked with such disposal have raised concern and even conscientious objection to the practice of letting controlled substances enter the wastewater system [23]. In a survey of hospice home care nurses, 55% said they always or often flushed the unused controlled substances, despite their own doubts about the environmental safety of such practice [24]. A study from Taiwan found appreciable amounts of controlled substances in the wastewater treatment plants near five hospital effluents and demonstrated that these substances eventually reach natural rivers, where they pose a significant risk to aquatic and human life [25]. In a study of the Great Lakes Basin in the US, detectable concentrations of a number of pharmaceuticals were found in 34% of water samples, but because of no national standards or guidelines, quantitative assessment could not be carried out [26].

Healthcare organizations face special environmental challenges. They have an unpredictable waste stream with a thousand or more potential pharmaceutical products in a variety of formulations (pills, patches, liquids, syringes, nebulizers, and so on). Few healthcare organizations have environmental experts on staff and most clinicians are not trained in handling hazardous waste. Thus, the professionals tasked with handling and discarding unused pharmaceuticals may not have a clear understanding of what can safely be disposed in the sewage and what constitutes an environmental hazard.

In addition to the federal EPA, states also have their own environmental authorities. Thus, hospitals and other organizations disposing of controlled substances must navigate what can be a network of regulations, all of which are subject to change.

In a study of two hospitals in Albany, New York, wasted controlled substances entering the water system were analyzed with the most dangerous substances for aquatic life identified as acetaminophen and codeine, acetaminophen and hydrocodone, acetaminophen and oxycodone, alprazolam, diazepam, fentanyl, midazolam, and testosterone. Morphine, ketamine, oxycodone, and zolpidem were less toxic but also of environmental concern [22]. In this study, midazolam, acetaminophen, codeine, and fentanyl were 87.5% of all drugs wasted [22]. Thus, the most dangerous controlled substances were exactly the ones entering the water system.

While it may be thought that water treatment plants are able to adequately remove controlled substances from the water, the fact is that sewage treatment plants are not designed to remove all of the pharmaceuticals in the water they receive, with the result that many pharmaceuticals pass through water treatment relatively unchanged [27,28].

Patient safety concerns

Drug diversion in the healthcare system may involve diluting controlled substances or diverting the patient’s dose so that the patient receives less medication than prescribed [29]. This may also be accomplished by dispensing the prescribed amount for the patient but then “wasting” a portion of it without actually disposing of it. (The allegedly wasted amount is then diverted.) These practices can result in substandard care, even agonizing pain for the patient. In other cases, drug diversion in a healthcare system may divert the controlled substances entirely from the patient, that is, a healthcare worker may dispense the drug, divert or consume it, and then state that the controlled substance was administered to the patient. Reports of such events appear in the literature [11]. Since such events may not be noticed by the patient, this situation is probably under-reported. Tampering with controlled substances by healthcare professionals seeking to divert drugs can result in contamination of the syringe or adulteration of the drug, exposing the patient to other risks, such as blood-borne infection. When bagged controlled substances are pilfered and contents replaced by equal amounts of water, the use of unsterile tap water places the patient at further risk.

Patients are placed at risk when they are treated by clinicians who are trying to divert drugs or are using drugs on the job. Inebriated clinicians can make errors in judgment, fail to observe the patients in their care properly, become distracted or “checked out,” or dispense drugs improperly [30]. Such activities compromise patient safety and exposes the institution to malpractice liability. Even if the clinician does not use controlled substances while at work, a focus on drug diversion may take precedence over the more important task of patient care. In other words, a drug-diverting clinician may become more focused on how to pilfer a syringe of opioid medication than that the patient is struggling with postoperative pain [11].

Healthcare professional risks

One in 10 American healthcare professionals has some form of substance use disorder [31], and it is likely that many are never detected. While this rate is roughly the same as the general population, meaning that healthcare workers are not at elevated risk for substance use disorder, healthcare professionals often have easier access to controlled substances than the general population. Healthcare workers are more likely to have a use disorder involving a prescription rather than an illicit drug [32]. Since they may not ever buy street drugs or have a street dealer, it may be easier for them to evade detection by authorities. Thus, the full extent of healthcare professionals with active substance use disorder may be greater than current assessments.

Healthcare professionals who are able to divert drugs in the workplace expose themselves, their patients, and their institutions to great risk. At the very least, drug diversion exposes the clinician to loss of licensure, possible criminal prosecution, and malpractice claims. Clinicians with active substance use disorder who care for patients while under the influence not only put patients at risk, but may suffer themselves in the form of shame, guilt, or feelings of being out of control [30]. In the event that drugs are diverted from a specific patient and covered up by false charting, there can be further prosecution for altering medical records and billing fraud (charging the patient or payer with drugs that were never administered) [11].

A clinician who is actively diverting drugs on the job may expose other clinicians to risk, for example, by leaving contaminated needles in unexpected areas. Clinicians who use drugs on the job may be less responsive or cooperative with their colleagues and create potentially dangerous situations both for patient care and collegial support [11]. Healthcare professionals who divert drugs regularly violate institutional protocol and may put colleagues at risk if those working with the drug diverter are aware or suspicious of what is going on. This can pose an ethical dilemma for some clinicians as they wonder if they ought to report such behaviors or just ignore them.

The institution also suffers when healthcare professionals divert drugs: they lose revenues from the drugs, become exposed to liability on many levels, and may have staff members who are frequently absent or perform their job at dangerously substandard levels [11]. For example, if a healthcare worker with a provable controlled substance use disorder harms a patient in his or her care, the institution, as well as the healthcare professional, may be held liable in civil litigation [11]. Morale on the clinical team may suffer when the staff recognizes a drug diverter who is able to “get away with it” without repercussions from the employer, particularly if they start to resent having to cover for a drug user. When drug diversion is merely suspected, the institution may be obliged to launch an investigation which is costly in time, money, and resources and distracts from the organization’s main mission [11].

Finally, healthcare workers who divert controlled substances for their own use are at risk for morbidity and mortality, the same as street drug users, although many healthcare workers have a sense that their greater medical knowledge makes them less vulnerable.

DEA final rule

The United States Disposal of Controlled Substances Act of 2010 (also known as the Disposal Act) went into effect on a federal level on September 9, 2014 [33]. This was largely an effort to regulate the appropriate disposal of unused controlled substances held by private individuals or organizations [17]. In this ruling, DEA has defined reverse distributors and authorized collectors, and sets forth guidance for long-term care facilities.

Long-term care facilities must rely on a third party, typically a pharmacy or hospital with an onsite pharmacy, to manage their controlled substance disposal although they typically maintain a collection receptacle on site. They may now store inner liners that have been removed from the collection receptacle and sealed onsite in a secure cabinet with a lock for up to three business days, if necessary. When these inner liners are picked up, they must be transferred by a common or contract carrier directly to the reverse distributor or distributor. In the past, the disposal of controlled substances at long-term care facilities had to be witnessed by two employees who documented the event. That is still permitted, but the long-term care facility now has the option of designating one supervisor-level employee, such as a charge nurse, to witness the removal of the waste [33].

New EPA hazardous waste regulation

The EPA issued regulations in August 2019 that prohibited the flushing or sewering of hazardous pharmaceutical waste. Controlled substances on the EPA list are exempted, for example: chloral hydrate, phentermine, fentanyl sublingual spray, testosterone gels, diazepam injectable, and paraldehyde. The EPA defines hazardous pharmaceuticals as those medications listed on the Resource Conservation and Recovery Act (RCRA) list or those having certain hazardous characteristics [34]. To satisfy the conditional exemption, the hazardous waste pharmaceutical must not be sewered, must be handled in compliance with DEA regulations, and must be destroyed by a method that meets the DEA's non-retrievable standard for destruction, i.e., incineration [34]. Also, this exemption extends to the household waste pharmaceuticals collected in take-back events. It is important to note that non-hazardous and hazardous pharmaceutical waste may be co-mingled in impermeable, non-reactive, and structurally sound containers until pick-up. Thus, all containers must be properly labeled as hazardous waste and include the container's start date.

Controversies about controlled substance waste

Guidance about controlled substance waste appears primarily in the form of regulations from multiple organizations, and this fragmented approach makes it challenging for healthcare facilities and other organizations to navigate the rules to assure their compliance, as well as to build practical and appropriate procedures into their workflow. No organization has issued definitive, specific guidance in the sense of a protocol. While this gives organizations a degree of flexibility, it can also make it challenging to set up a compliant sequence of steps that is robust enough to resist diversion and meet environmental and legal requirements while still being economically feasible.

A further complication to controlled substance waste is novel product formulation. For example, controlled substances are sometimes delivered in a transdermal system (buprenorphine, fentanyl) and the disposal of the used patch can be controversial. Manufacturers and even the FDA allow the patch to be folded (adhesive sides together) and flushed [5]. Most transdermal systems are designed so that only about half of the drug is delivered through the skin and a substantial amount resides in the patch even after use [35]. Flushing used patches may actually put a larger than expected quantity of controlled substances into the water system. For that reason, it may be more environmentally sound to use an agent to deactivate the drug and then dispose of the depleted patch [35].

Discussion

In the absence of formal protocols or guidelines, there are a number of steps that hospitals, clinics, and other organizations can take to help reduce drug diversion and assure proper controlled substance disposal.

**Table 3:** Practical advice for organizations handling controlled substances.

Advice	Rationale	Comment
Do not allow clinicians access to the controlled substances disposal receptacle if they have a bag, backpack, purse, or another container with them	It is easy to divert a drug at the point of disposal	Drug diversion here is difficult to detect if the clinician documents that the drug is put into the receptacle but then hides it
Set par levels of controlled substances at suitable but low levels	Avoid having high volumes of controlled substances available	Large amounts of controlled substances can make it easier to camouflage diversion
Controlled substances should be available in low-dose formulations	Using multiple low-dose products prevents waste	Instead of a 10 mg syringe of morphine, 2 mg syringes of morphine can be stocked
Reconcile controlled substances frequently	Exposes potential diversion or problems	Investigate as soon as possible; the more time elapses, the harder diversion can be to prove
Do not discard vials or syringes with controlled substances in them	If such items must be discarded, draw out the unused controlled substance with a witness present and waste the substance	This step (removing the contents of the vial or syringe) requires the quantity be measured, the event documented, and a witness signing off
Audit controlled substance documentation frequently	The longer the interval between removing a controlled substance from inventory and dispensing, the more likely diversion can occur	Compare pain medication dispensing to pain intensity reports and note if pain intensity is higher with one clinician more than others
Do not assume that irregularities or such events are proof of diversion	Be mindful and check on these events but use them as a starting point for a conversation rather than an accusation	Investigations may be necessary not just for drug diversion but to assure all staff and hospital administration that the subject of controlled substance diversion is taken seriously

**Figure 1:**
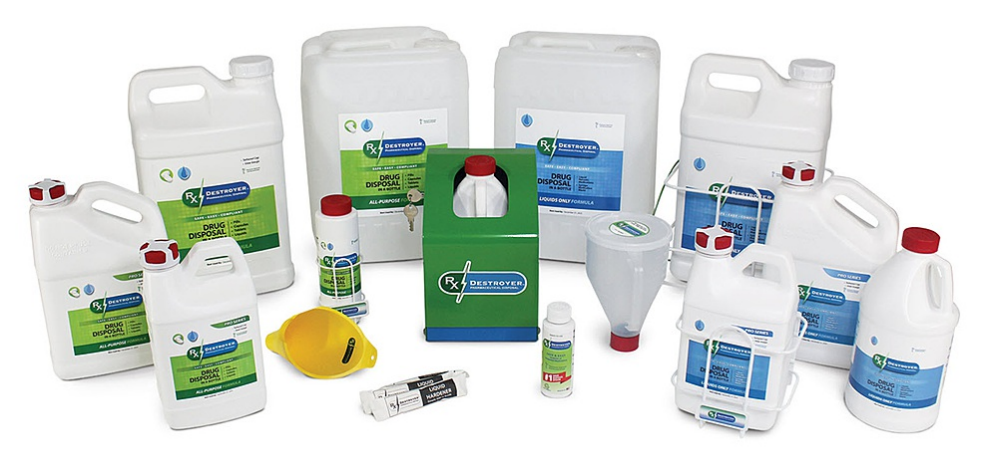
An example of a leading commercially available product for rendering controlled substances non-retrievable and allowable for landfill waste Photograph courtesy of RxDestroyer™, Burlington, Wisconsin; used with permission.

Compliance issues can be resolved with the use of certain commercial products that adhere to both DEA and EPA regulations. Commercial products are available that can chemically neutralize pharmaceuticals, including controlled substances, without water or incineration, rendering the destroyed substances landfill-friendly. Products such as RxDestroyer™ (Rx Destroyer - Drug Disposal System, Burlington, Wisconsin) use a proprietary liquid and activated carbon to render the substances non-retrievable (See Figure 1). While such systems have limitations, for example, they cannot be used for the destruction of vials, syringes, glass, and so on, they provide a viable and cost-efficient solution for many organizations tasked with the disposal of controlled substances. This provides a solution that protects the environment, is easy to use, and essentially renders the active-controlled substances non-retrievable and unusable. Ultimately, for optimal safety and best practices, all controlled substance waste stored in holding containers should be sent off for eventual physical destruction, i.e., incineration. This can be accomplished by utilizing the services of a licensed waste management company, which is responsible for disposing of all healthcare-related waste. In the interim, an easy-to-use disposal system makes it easier for clinicians to be compliant and protects the institution, its staff, and patients. Thus, controlled substance waste generated in healthcare facilities can be stored in holding containers, which discourage diversion until they are ultimately incinerated. This process, in effect, would comply with the DEA final rule for controlled substance waste and ensure that the healthcare facility is in regulatory compliance. 

## Conclusions

The disposal of controlled substances is a crucial matter for public health with many key stakeholders and a network of government organizations tasked with overseeing this process. The result has been a confusing and sometimes contradictory array of rules and regulations that are slowly being sorted out. Private industry has assisted by developing safe, user-friendly, and cost-effective products to help render controlled substances non-retrievable in such a way that they meet DEA and EPA standards. The organizations and entities such as hospitals, surgical centers, dental offices, and so on are all potential repositories for controlled substance waste. In this regard, there should be a safe, secure, and effective mechanism for dealing with this accumulated waste. It is the onus of healthcare facilities and practitioners, which utilize controlled substances to have effective policies and procedures in place for handling their waste so as to protect the environment, maintain public and patient safety, discourage diversion, and thus avoid regulatory scrutiny. Patients who are burdened with having to dispose of their own controlled substance waste need the guidance of healthcare professionals to ensure that they are taking the proper steps to maintain safety, comply with regulations, and satisfy environmental concerns. 
